# School Violence towards Peers and Teen Dating Violence: The Mediating Role of Personal Distress

**DOI:** 10.3390/ijerph18010310

**Published:** 2021-01-04

**Authors:** Sonsoles Valdivia-Salas, Teresa I. Jiménez, Andrés S. Lombas, Ginesa López-Crespo

**Affiliations:** Department of Psychology and Sociology, University of Zaragoza, 44002 Teruel, Spain; sonsoval@unizar.es (S.V.-S.); slombas@unizar.es (A.S.L.); glopezcr@unizar.es (G.L.-C.)

**Keywords:** school violence, teen dating violence, personal distress, mediation, adolescence

## Abstract

School violence towards peers and teen dating violence are two of the most relevant behaviour problems in adolescents. Although the relationship between the two types of violence is well established in the literature, few studies have focused on mediators that could explain this empirical relationship. We departed from the evidence that relates anger, emotional distress and impaired empathy to teen dating violence and juvenile sexual offending, to explore the role of personal distress, i.e., a self-focused, aversive affective reaction to another’s emotion associated with the desire to alleviate one’s own, but not the other’s distress; as a possible mechanism linking school violence towards peers and teen dating violence in a sample of Spanish adolescents. We also explored the prevalence of emotional and physical teen dating violence, both occasional and frequent, and the differences between boys and girls. A total of 1055 adolescents (49.2% boys and 50.8% girls) aged between 11 and 17 years (*M* = 14.06, *SD* = 1.34) who had had at least one romantic relationship within the last year, completed measures of school violence towards peers, teen dating violence, and personal distress. Statistical analyses revealed that occasional and frequent teen dating violence (both physical and emotional) was more frequent in girls than in boys, and that personal distress functioned as a partial mediator, with an overall model fit higher for boys than girls: in boys, partial mediation occurred for both physical and emotional teen dating violence; in girls, partial mediation occurred only for physical violence. The interpretation of the results is tentative given the novel nature of the study, and points to the evidence of the emotional costs of school violence and the importance of emotion and behavior regulation to undermine the social costs of personal distress.

## 1. Introduction

Violence towards peers at school settings and towards a partner in intimate relationships are two of the most relevant behaviour problems in adolescents. Both are frequent (at least one in three adolescents have suffered some peer or dating violence) and have important negative consequences for psychosocial adjustment with repercussions on the person’s integral development. For these reasons, both are considered two serious public health problems [1,2].

### 1.1. Teen Dating Violence

Teen dating violence (TDV) refers to a wide range of partner-directed harmful behaviours among adolescents. TDV may be psychological (e.g., emotional manipulation), physical (e.g., shoving, slapping, kicking), and/or sexual (e.g., forced sexual activity) [3]. Compared to research with adult partner violence, research on TDV is more recent, also in Spain [4]. Nowadays, the prevalence rates of TDV in international studies range from 20.2% to 77% for emotional violence, from 9.8% to 37.7% for physical violence, and from 7.4% to 15.3% for sexual violence, with similar prevalence rates for boys and girls, except for sexual violence, of which girls are more likely to be victims (for a review see [5]). In addition to these alarming figures, the most frequent forms of TDV (i.e., emotional violence and control tactics) are related to poor emotional adjustment and a higher probability of suicidal ideation on the side of the victim [6,7], and may be linked to later violence within adult relationships [8,9]. For these reasons, delving into the study of the factors associated to TDV (for a review of the risk factors associated to TDV see [10,11]) becomes the target of researchers and educators interested in the early detection and prevention of this pattern of behaviour.

### 1.2. School Violence towards Peers

Among the different acts involving school violence (i.e., aggression towards school staff, property damage, vandalism in the school campus and bullying), the current study focused on school violence towards peers (henceforth, school violence or SV), that is, on behaviours directed towards other classmates with the intention to cause harm. These acts can manifest as physical (pushing, hitting, kicking, shoving, etc.), verbal (name-calling, taunting, threatening, etc.) or relational violence (spreading false rumours or malicious gossip, excluding from activities or withdrawal of friendships). The first two forms of violence have been labelled as “direct or overt”, and the third as “indirect or relational”. To understand the relevant negative consequences of these behaviours in adolescents -for a review see [12], it is important to note that the perpetrator usually acts with the desire and intention to dominate and to exert control over the other person [13]. Research in the last two decades has identified SV as a serious problem in European [14], North American [15], Latin American [16], and Asian countries [17]. International data indicate that prevalence rates vary greatly among countries. For example, in a study conducted with adolescents from 43 countries (Europe, USA and Canada) by the World Health Organization (WHO) [18], the rates of SV perpetration varied from 1% to 36%, and the rates of SV victimization between 2% and 32%, depending on the country and the way school violence was assessed [19]. García-García et al. [20] conducted a systematic review of 32 studies assessing the prevalence of SV in Spanish samples, and it yielded an overall rate of 11.45%. As for gender, the rates of SV perpetration and victimization are consistently higher for boys when compared to girls in cross-cultural surveys [21]. Beyond its magnitude, the relevance of SV relates to the severity of its outcomes at the personal (e.g., anxiety, depression, low self-esteem and life satisfaction), family (e.g., conflict, communication problems, low affective union), and school level (e.g., negative attitudes toward school, low academic achievement, school drop-out) for the victims and the perpetrators [12,22,23]. Focusing on perpetrators, although SV shows some benefits in the short term such as social reputation and self-esteem [24,25], it also has a negative impact on the psychological adjustment of the perpetrator such as high perceived stress, depressive symptomatology, and low life satisfaction [23,26,27]. Furthermore, it has been related with an increased likelihood of violence perpetration later in life [28]. Still, the proximal consequences of SV in the context of the first intimate relationships are not clear.

### 1.3. Relation between School Violence towards Peers and Teen Dating Violence

Several studies have found consistent relationships between SV and TDV [29,30,31,32]. In the case of victimization, there is evidence that when it is experienced in one context (e.g., at school) it is more likely that it will be experienced in other contexts (e.g., intimate relationships) through psychological risk factors such as perceived loneliness and low life satisfaction [33]. As for perpetration, Cava et al. [26], for instance, showed that TDV had medium-size correlations with overt SV and medium to low-size correlations with relational SV. As a matter of fact, some authors have argued that SV and TDV are related to the same control-submission schema and could be part of a general pattern of antisocial behaviour in specific groups of adolescents [32,34,35]. All these results support the idea of a transcontextual usage of violence in significative relationships (peers and partners) during adolescence.

Although the relationship between SV and TDV perpetration is well established in the literature (see [36], for a systematic review and meta-analysis), research on this topic is still scarce, including research on the mediators that might explain this empirical relationship. For instance, considering the developmental course of different types of aggression during adolescence, Cutbusch et al. [37] explored the mediating role of sexual harassment but the results did not support their hypothesis because both SV and sexual harassment predicted later TDV. More promisingly, and departing from the evidence that emotional distress, and particularly anger and lack of emotional self-regulation, predicted later dating violence perpetration [38], Foshee et al. [39] demonstrated that anger was a mechanism linking SV perpetration and TDV perpetration.

The role of anger in dating violence perpetration has been also explored in relation with gender-role discrepancy and the associated distress. Gender roles set socially constructed expectations and norms about appropriate male and female behavior and the culturally acceptable dynamics between males and females. According to such gender roles, males are expected to be tough and dominant, with violence being an effective way to demonstrating these qualities. Discrepancy stress arises when a male believes that he is, or believes he is perceived to be, insufficiently masculine. There is evidence that experiencing discrepancy stress in early dating relationships leads to stereotypical masculine behaviour (e.g., aggression, risky sexual behavior) and to interpretations of interpersonal interactions in intimate relationships as a threat to masculinity and hence to violence, as a way to demonstrating and validating masculinity to self and others [40,41].

Aiming at further exploring possible variables intervening in the relation between SV and TDV, in our study we depart from the evidence on the relation between empathy and violence, and its counterpart, prosocial behaviour. Such evidence is inconclusive [42], partly because of the complex nature of empathy itself, which has promoted the emergence of numerous models and theories about what empathy is, and its relation with other constructs and outcomes [43].

### 1.4. Violence and Empathy: The Role of Personal Distress

The multidimensional model of empathy by Davis [44], for instance, distinguishes between cognitive empathy (perspective taking and fantasy) and affective empathy (empathic concern –EmpCon- and personal distress –PD-). Cognitive empathy is the ability to understand how others feel, whereas affective empathy entails the vicarious experience of others’ feelings [45]. If above some minimal threshold, such vicarious experience of other’s feelings may turn into EmpCon, PD, or both [46,47]. While EmpCon consists of feelings of sorrow or concern for the other, PD is a self-focused, aversive affective reaction to another’s emotion associated with the desire to alleviate one’s own, but not the other’s distress. Accordingly, EmpCon tends to be positively related to helping others, whereas PD tends to be negatively or unrelated to prosocial behaviors [47,48]. Considering that adolescence is characterized by narcissism, the exaggeration of gender-specific roles (males’ control and females’ submission), the mystification of romance and the lack of experience in dating relationships [1], we would expect that dating relationships could be a relational context relevant for experiencing PD.

A number of studies have explored the actual relation between low empathy, both cognitive and affective, and different types of violent behaviour. For instance, Jolliffe & Farrington [49] showed that low total empathy related to overt school violence in males and to indirect school violence in females, being the affective but not the cognitive components what most contributed to this relation. Similarly, Gini et al. [50] showed that low empathy responsiveness related to SV perpetration but only in boys. More recently, Euler et al. [51] found that both cognitive and affective empathy were negatively related to proactive aggression. The negative relation between empathy and TDV has also been reported [11]. All in all, as reviewed and reported by Van Noordeen et al. [52], deficits in cognitive empathy have been mostly reported in offending, and deficits in affective empathy have been mostly observed in antisocial behaviour, adolescent aggression and delinquency, and SV perpetration [23,27] (for further evidence see [53,54,55,56]).

While the evidence is large for total empathy score and total cognitive and affective total scores, the evidence of the relation between violence and particular components of empathy is scarcer. Especially relevant to this paper is the evidence of the role exerted by PD, one of the components of affective empathy. This evidence has been mainly collected with sex-offending delinquent juveniles, and shows, for instance, that the acceptance of sexual violence correlates positively with PD [57], and that juvenile sex offenders score significantly higher on PD than non-sex-offending delinquents and non-delinquents [58,59]. Still, this scarce evidence calls for further research [60].

Considering that (1) SV has emotional costs; (2) emotional states such as anger, emotional distress and personal distress relate to TDV and juvenile sexual offending, and (3) teen dating relationships are a source of stress associated to the lack of experience, the exaggeration of gender roles, and gender-role discrepancies; we might hypothesize that PD could be one mechanism linking SV and TDV.

### 1.5. The Present Study

According to previous literature, in the present study we seek to (1) analyze the prevalence of emotional and physical TDV, both occasional and frequent; (2) determine whether PD mediates the relationship between school violence and TDV; and (3) analyze differences between boys and girls; in a sample of Spanish adolescents. We expect to find the mediating role of PD and no gender differences in the mediation relationships among the study variables.

## 2. Materials and Methods

### 2.1. Ethics Approval

The present research was carried out in compliance with the ethical standards required for research with human beings, respecting the basic principles included in the Declaration of Helsinki and the code of good research practices of the host university. The study was approved by the Research Ethics Board of the Health Department of the corresponding Government (title of the research: Violence, Gender, and Psychological Inflexibility in Adolescence. Protocol number: PI20/122).

### 2.2. Participants

The sample included a total of 1065 adolescents (participation rate of 99%) who had had at least one romantic relationship within the last year (49.2% boys and 50.8% girls), aged between 11 and 17 years (*M* = 14.06, *SD* = 1.34), and enrolled in 11 Secondary Compulsory Education schools across different regions of Spain. Student distribution by academic grade was the following: 18.2% were enrolled in the 1st grade of secondary studies, 28.9% in the 2nd grade, 26.4% in the 3rd grade, and 26.5% in the 4th grade.

### 2.3. Instruments

#### 2.3.1. Dating Violence. Conflict in Adolescent Dating Relationships Inventory (CADRI)

This scale, developed by Wolfe et al. [61], was designed to assess “multiple forms of abusive behavior that may occur between adolescent dating partners” [4]. The original scale consists of 25 items. Responders rate how often they have had arguments or conflicts with their couples over the last 12 months, on a 4-point scale ranging from 1 (never) to 4 (often). The scale has a structure of five factors, representing five different types of abuse—physical, sexual, threatening, relational, and emotional or verbal. However, the Spanish validation developed by Fernández-Fuertes et al. [4], showed that only three factors maintained optimal reliability: emotional violence, with 10 items (*r* = 0.79); physical violence, with four items (*r* = 0.76) and relational violence, with only three items (*r* = 0.73). In the present study, we used the subscales of emotional violence (e.g., “I blamed her for the problem,” “I spoke to her in a hostile or mean tone of voice”) and physical violence (e.g., “I pushed, shoved, or shook her”), which showed optimal reliability in our data (between 0.70 and 0.80 for both boys and girls).

#### 2.3.2. School Violence 

This scale developed by Little et al. [62] (bidirectional translation into Spanish, using the parallel back-translation procedure of Brislin [63]), includes 25 items that assess participation in aggressive behavior towards peers at school over the last 12 months. It is rated on a 4-point Likert-type scale ranging from 1 (never) to 4 (always). The scale evaluates six dimensions of aggression within two types of aggressive behavior—overt or direct, and relational or indirect— and three functions of violence—pure, reactive, and instrumental—. These dimensions are: pure overt (e.g., “I’m the kind of person who hits, kicks, or punches others”), reactive overt (e.g., “If others make me upset or hurt me, I often put them down”), instrumental overt (e.g., “I often threaten others to get what I want”), pure relational (e.g., “I’m the kind of person who says mean things about others”), reactive relational (e.g., “If others have threatened me, I often say mean things about them”), and instrumental relational (e.g., “To get what I want, I often ignore or stop talking to others”). In the present study and considering the difficulty of working with such a high number of items, all items were summed up to generate six composite variables that fed the School Violence construct in the measurement model. The Cronbach alpha values obtained in previous studies with Spanish adolescent samples ranged between 0.72 and 0.87 [23,64]. In our sample, Cronbach’s alpha of the global scale using these composites was 0.84 for boys and 0.83 for girls.

#### 2.3.3. Personal Distress 

To evaluate Personal Distress, we used one of the four subscales included in the Interpersonal Reactivity Index (IRI) [65,66]. This subscale contains 7 items about empathetic distress (e.g., “I sometimes feel helpless when I am in the middle of a very emotional situation”). Responders rate how well the items describe them on a 5-point scale ranging from 1 (does not describe me well) to 5 (describes me very well). Same as in the original scale, there were two reversed items in our questionnaire: “When I see someone get hurt, I tend to remain calm”, and “I am usually pretty effective in dealing with emergencies.” These two items introduced reliability problems and were hence removed from the analysis. The question of how reversed items affect the reliability of the scales has been widely discussed. For instance, some researchers have stated that it is a questionable practice, and others have eliminated them from their scales after some empirical and logical analyses [67]. In our study, after removing these two items, the Cronbach’s alpha for PD was 0.75 for boys and 0.66 for girls.

### 2.4. Procedures

The present data were collected as part of a larger study on dating violence in adolescents. A letter with a summary of the research project was sent to the selected schools as a first step and we contacted the principal of each school to explain the purpose of the research and to request permission to carry out the study. We then requested parents’ and guardians’ consent for the children to participate in the study (only 1% did not give consent). We explained the goals of the study to the students and informed them that participation was voluntary and anonymous. Measures were collected in their respective classrooms during a regular class. At least one qualified researcher (with a Ph.D.) was present during the administration of the instruments to provide students with the necessary support. The order of administration of the instruments was counterbalanced in each classroom and school.

### 2.5. Statistical Analysis

First, descriptive statistics (means and standard deviations) and correlations were computed using SPSS (version 26.0, IBM, Armonk, NY, USA). Spearman’s correlations, appropriate for the ordinal nature of our variables, were estimated. After descriptive statistics, multivariate inferential analyses were conducted using structural equation modeling (SEM) through Mplus 8.4 software (Muthén & Muthén, Los Angeles, CA, USA) [68]. These analyses were carried out for boys and girls separately because preliminary analyses revealed a lack of invariance of the measurement model as a function of gender. Indeed, Davis [44] and Wolfe [61] had already informed about relevant differences between males and females in their responses to PD and CADRI sub/scales.

Given the ordinal nature of the output variables, the analysis was performed using a robust weighted least square estimator and robust errors estimations (WLSMV in Mplus terminology), which is the most appropriate approach with categorical indicators and a sample of 200 or more units [69]. However, this change has implications in the estimation and interpretation of the coefficients with respect to those from models estimated by maximum likelihood. In this case, the model is considering probit instead of linear slopes. The results are more similar to linear ones by reporting standardized results, because they are estimated with an underlying continuous variable, but categorical items may, though, show smaller coefficients due to being less reliable indicators [68].

Statistical significance was set at *p* = 0.05 throughout analyses. We performed a SEM in two steps. First, a confirmatory factor analysis was conducted to obtain a measurement model and hence, to assess the psychometric properties of the instruments. Afterwards, we conducted a simple mediation analysis projected on our two outcome variables, namely physical and emotional dating violence perpetration. This was done by following the causal steps approach [70], according to which a variable must meet the following conditions to be considered a mediator: (1) the fit of the overall model when the dependent variable, C, is regressed on the predictor, A, has to be good (A-C model), and the A-C path coefficient has to be significant; (2) the fit of the overall model when the dependent variable, C, is regressed on the mediator, B, and the mediator is simultaneously regressed on the predictor, A, has to be good (A-B-C model), and the A-B and the B-C path coefficients have to be significant; (3) there must not be a significant improvement in fit when comparing A-B-C model when the A-C path coefficient is unconstrained (Unconstrained A-B-C Model), respective to when this path coefficient is constrained at zero (Constrained A-B-C Model).

In order to obtain a significance test of the comparison of these two structural models (i.e., constrained and unconstrained), a Chi Square Difference Test was performed using the DIFFTEST option in Mplus. The satisfaction of this condition proves complete mediation. If this condition is not met and the existence of partial mediation is to be assessed, then a significance test of the indirect effect should be performed. In our study, we evaluated this condition, referred to as condition 4 independently of the result in the condition 3, since it provides an index of the magnitude of the mediational effect. When condition 3 was satisfied, indirect effect was calculated on the Constrained A-B-C Model, otherwise the indirect effect was calculated on the Unconstrained A-B-C Model. Since the satisfaction of condition 3 indicates a non-significant A-C path coefficient, the calculation of the indirect effect in the Constrained A-B-C model should lead to a model with higher statistical power, respective to the Unconstrained A-B-C model, as the result of having two less parameters. In order to perform the test of the indirect effect, its confidence intervals were calculated using the bootstrap method with 2000 samples.

Additionally, path coefficients magnitudes were accompanied by their bootstrapping confidence intervals. A model had good fit when Root Mean Square Error of Approximation (RMSEA) was higher than the confidence interval and Standardized Root Mean Square Residual (SRMR) values were lower than 0.08, and when Comparative Fit Index (CFI) value was higher than 0.90 [71]. As usual, a Chi-square test for model fit was reported but not to evaluate the fit of the models due to its sensitivity to large sample sizes.

## 3. Results

### 3.1. Descriptive Analysis

Among the participants, 58.8% of boys (sometimes 53.4% and often 5.4%) and 73.5% of girls (sometimes 62.3% and often 11.2%) stated having perpetrated at least one kind of emotional violence towards their partner in the last 12 months. Of these, 28.0% of the boys and 43.2% of the girls affirmed that they exerted such violence using three or more different methods. The more usual types of emotional TDV among boys were “I did something to make her feel jealous” (32.3%) and “I said things just to make him/her angry” (31.1%). Among the girls, the more usual types of emotional TDV were “I did something to make him feel jealous” (49.8%) y “I accused him of flirting with another girl” (39.4%). The most frequent type of physical TDV in both genders was “I threw something at him/her” (14.7% in boys and 20.6% in girls). The other three items assessing physical TDV obtained responses lower than 10%, although always higher among girls.

Table 1 shows differences between boys and girls in the two types of TDV. In all cases (types and frequency of violence), the data yielded a higher percentage for girls. Pearson’s chi-square analyses indicated that these gender differences were statistically significant in both physical (χ^2^ = 31.25; *df* = 2; *p* < 0.01; Cramér’s V = 0.172) and emotional TDV (χ^2^ = 12.37; *df* = 2, *p* < 0.01; Cramér’s V = 0.108).

The descriptive statistics and bivariate correlations for our observable variables are presented in Table 2 (Table 2 presents significant values, for a complete table see Table A1 in the Appendix A). The correlations between the items of the same factor were positive and significant, with *r* ranging from 0.12 to 0.56, *p* < 0.01. In general, most of the bivariate correlations between the observed variables of SV, PD and TDV were significant at 0.05 level except for some PD items: for example, PD1 (“In emergency situations, I feel apprehensive and ill-at-ease”) only correlated with one indicator of physical TDV in girls and three indicators of emotional TDV in boys; however, PD4 (“I tend to lose control during emergencies”) correlated with the majority of the emotional TDV indicators in girls; and PD5 (“When I see someone who badly needs help in an emergency, I go to pieces”) correlated with the majority of the emotional and physical TDV indicators in boys. All the significant bivariate correlations between the observed variables were positive.

### 3.2. Structural Equation Modeling

#### 3.2.1. Measurement Model

The fit indices for the measurement model were as follows: for boys, χ^2^ = 308.9, *df* = 269, *p* < 0.05, CFI = 0.98, TLI = 0.98, RMSEA = 0.02 (90% CI 0.00–0.03), SRMSR = 0.06; for girls, χ^2^ = 344.12, *df* = 269, *p* < 0.01, CFI = 0.96, TLI = 0.96, RMSEA = 0.02 (90% CI 0.02–0.03), SRMSR = 0.07. Values of the fit indices showed that the measurement model had an adequate fit (see Table 3 for factor loadings, Cronbach’s alpha, McDonald’s omega coefficients and covariances between latent variables on the measurement model).

It is relevant to note that the relation between SV and PD, as latent variables, was more than double in boys than in girls (0.41 and 0.18, *p* < 0.01, in boys and girls, respectively).

#### 3.2.2. Mediation Model

**Figure 1:**
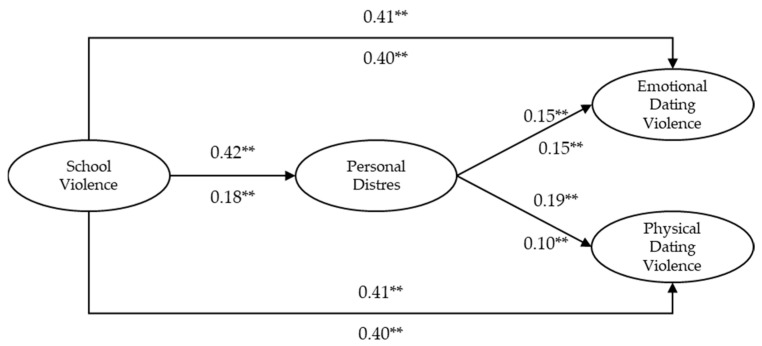
Unconstrained A-B-C model with path coefficients and statistical significance. ** *p* < 0.01. β values for boys are above the path and β values for girls are below the path.

The role of PD as a mediator between SV and TDV was examined. The four conditions required by the causal steps approach to evaluate this mediation are presented in Table 4 for boys and in Table 5 for girls. For a visual inspection of the mediational model, see Figure 1.

All the conditions were satisfied in boys, except for condition 3. That is, the Loglikelihood Chi Square Difference Test was statistically reliable, indicating that PD functioned as a partial mediator. The direct effect between SV and emotional and physical TDV in the A-C model was significant (β = 0.47 and β = 0.49, respectively, *p* < 0.01) and, although its magnitude decreased slightly after entering PD as a mediator in the Unconstrained A-B-C model (β = 0.41 *p* < 0.01 for both types of TDV), it remained significant. The indirect effect between SV and emotional and physical TDV through the mediation of PD was small but significant for both types of TDV (β = 0.06, *p* < 0.05 and β = 0.08, *p* < 0.01, for emotional and physical TDV, respectively).

In a similar way, all the conditions were satisfied in girls, except for condition 3. That is, the Loglikelihood Chi Square Difference Test was statistically reliable, indicating that PD also functioned as a partial mediator. However, the overall fit of the models was worse in girls than in boys. The direct effect between SV and emotional and physical TDV in the A-C model was also significant although weaker than in boys (β = 0.42, *p* < 0.01 for both types of TDV). Furthermore, the magnitude of the direct effect after entering PD as a mediator in the Unconstrained A-B-C model barely changed (β = 0.40, *p* < 0.01 for both types of TDV). Accordingly, the indirect effect between SV and emotional and physical TDV through the mediation of PD was four times smaller than in boys, and it was only significant for physical TDV (β = 0.02, *p* < 0.05).

In sum, the analyses conducted revealed that PD worked as a partial mediator in the relation between SV and both types of TDV in the case of boys, and between SV and physical, but not emotional, TDV in the case of girls. For both, boys and girls, the direct effect between SV and TDV remained significant after the inclusion of PD in the equation.

## 4. Discussion

The main objectives of the present study were to analyse the prevalence of emotional and physical TDV in a sample of Spanish adolescents and to determine whether PD mediates the relation between SV and TDV. We expected to find evidence for the mediating role of PD and no gender differences in the relationships among the study variables. Our findings have yielded gender differences in prevalence of emotional and physical TDV but also in the pattern of relationships among the variables, showing evidence for a partial mediation of PD between SV and both types of TDV in boys, and between SV and only physical TDV in girls.

### 4.1. Prevalence of Teen Dating Violence

Regarding prevalence, our data show that occasional and frequent TDV (both physical and emotional) was higher in girls than in boys. This finding adds to the inconclusive evidence reported so far. For instance, Dosil et al. [1] has recently informed that data in Spain point to a higher percentage of male perpetrators, with percentages of perpetration ranging from 7.5% to 37.8%, compared to 7.1% to 14.9% for females. Esparza et al. [72], however, found a higher percentage for overall female perpetration in Spain, specifically for emotional and physical violence. But this finding extends to international samples as well. As reported by Capaldi et al. [73], a systematic review of the literature on gender differences in dating/intimate partner violence, including 170 articles with adult samples and 58 articles with adolescent samples, yields consistent results: women are slightly more likely than men to use one or more acts of physical aggression and to use them more frequently (see also [74]). All in all, the data is still inconclusive, and this may be the result of using different measuring tools, not isolating other sociodemographic risk factors, or the likelihood that girls/women tended to minimize victimization and maximize perpetration, whereas boys/men tended to minimize perpetration and maximize victimization, as gender-role discrepant behaviors [41,72].

### 4.2. The Role of Personal Distress as a Mediatior between School Violence towards Peers and Teen Dating Violence

We found evidence for the mediational role of PD between SV and TDV. This mediational effect was small and partial for both boys and girls, with a better overall fit in boys. The fact that the mediation was partial means that there is still an amount of variance in TDV that is directly related to SV. This is in line with the literature that has consistently related both forms of violence and that poses them as two topographies of the same class of aggressive, violent, or antisocial behaviors [32,34]. In order to apprehend the scope of the novel finding in the present study, i.e., the mediational effect exerted by PD, we will firstly discuss the effect of SV on PD, then we will proceed to discuss the observed effect of PD on TDV; and finally, we will go over the gender differences found in the mediational effect.

The observed effect of SV on PD adds to previous evidence on the negative psychological consequences of this type of violence, so that in addition to perceived stress, depressive symptomatology and low life satisfaction [23,24], now we know that SV perpetrators also experience PD. This finding is particularly relevant when considering the scarce evidence on the costs of SV in terms of impaired empathy. Indeed, empathy impairments have usually been regarded as risk factor for the display of general [75] and school [27,49] violence. But the evidence is scarcer when it comes to predict empathy as a function of SV perpetration [23]. To our knowledge, there is evidence, although not without controversy [76], that media violence usage reduces affective empathy over time [77] through a process of habituation of seeing others suffering. But this evidence may not be informative to the present study because of two reasons; on the one hand, a general measure of affective empathy is reported; on the other hand, media violence does not involve real humans. Impaired affective empathy usually encompasses both reduced empathic concern for others and increased PD. Especially relevant in the context of the present investigation is that PD is a self-focused, aversive reaction to another’s emotion, that, in fact, is associated with the desire to alleviate one’s own but not the other’s distress [49]. Hence, our data supports the idea that SV perpetrators feel distress towards their victims, a type of distress that makes them more likely to act aiming at reducing their own distress, but not the distress of their victims. In other words, it seems that the increased status and reputation that usually occurs with SV perpetration [78] does not come without a high psychological prize, i.e., increased self-focused and aversive PD.

Indeed, our results point to PD as one possible risk factor for TDV. And this adds to previous evidence on other sources of stress for TDV such as family and peer problems [10,11]. Future studies will elucidate the relative contribution of each possible source of dis/tress during adolescence on the occurrence of TDV. But for now, it seems relevant to bring to bear the evidence that poses the first romantic relations as sources of distress associated to the lack of experience in dating relationships, the mystification of romance, and the exaggeration of gender-specific roles [1]. The exaggeration of gender-specific roles may, indeed, lead to the so-called gender-role discrepancy and the associated distress, phenomena that have been mainly described in males. Males are expected to be tough and dominant, and when this does not occur, they may experience discrepancy stress. There is evidence that experiencing discrepancy stress in early dating relationships leads to stereotypical masculine behaviour (e.g., aggression, risky sexual behavior) and even violence, as a way to demonstrating and validating masculinity to self and others [40,41]. It might be the case that seeing the victim partner showing some kind of vulnerability (e.g., for being in need for help) somehow activated the perpetrator’s lack of management skills, gender-congruent attitudes or beliefs, even gender-role discrepancies, that could increase his PD to a point in which violence was the only way to alleviate his own distress. Considering the higher prevalence of TDV among girls observed in the present and previous studies, further research will elucidate how discrepancy stress, which has been defined in males, may apply to female PD and TDV perpetration.

### 4.3. Gender Considerations

We found gender differences in the mediating role exerted by PD that deserve some considerations. As it has been noted before, the mediational effect was small but significant and four times bigger for boys than for girls in the case of physical TDV. In the case of emotional TDV, the indirect effect of SV through the mediation of PD was only significant in boys but not in girls. The latter finding might be due to the weak relationship we found between SV and PD in girls. In fact, this relationship was more than double in boys than in girls. In other words, in the case of girls, PD is not so nurtured by SV, and other variables, unexplored in the present study, would be explaining the observed PD.

When it comes to the role of gender on intimate partner violence perpetration rates and motives, the literature is large and terribly inconclusive e.g., [79]. It was not our purpose to cover this controversy but rather to try to understand why SV perpetration does not relate to a higher PD for the victim in girls as much as in boys; and why girls’ PD for their victims influences only physical but not emotional TDV perpetration. As for the first question, literature on the changes observed in gender socialization within the last decades may be enlightening. A recent review [80] highlights how changes in demographics, the global media and gendered economic opportunities have transformed the gender socialization process, with evident results in gender norms and identities. Over the last decades, a number of programs have been implemented specifically aimed at empowering girls with information and skills to challenge norms (including gender-role norms), and at changing boys and men gender-superiority attitudes and beliefs. Beyond the unquestionable benefits of this movement at a macro scale, the consequences at the relational level in the first romantic relationships might be what we found in our study. Girls, who according to normative gender stereotypes, are supposed to be caring, do not seem to show much care for their victims, so that seeing them in need for help does not elicit much PD. On the contrary, boys, who according to normative gender stereotypes, are supposed to be tough, get affected by their victims’ suffering. The worrisome side of this changing roles is that it might come parallel with: (1) in boys, an increase in PD for the victim, which is a self-focused type of distress which does not facilitate prosocial behaviour; and (2) in girls, an increase in the prevalence of female violence perpetration e.g., [72,73,74]. Future research will elucidate the benefits of considering other variables which could modulate the effect of gender-related attitudes and beliefs and the associated PD on actual behaviour, such as perspective-taking [47] and psychological flexibility [81,82].

As for the second question, i.e., why girls’ PD for their victims influences only physical but not emotional TDV perpetration, our interpretation can only be tentative due to the scarce evidence on physical TDV perpetrated by females e.g., [83,84], and also to the lacking literature on the role of PD on TDV. The literature on the motives for different types of TDV might be informative. There is evidence, for instance, that women are more likely than men to commit physical violence that is motivated by self-defense, fear [83], anger and stress [84]. It could be that, as compared to other types of violence, physical violence in female related to the self-preservation instinct that is typical in situations of stress associated to danger. If this was the case, our findings would point to the possibility that PD in female adolescents related to more instinctive or impulsive forms of violence (i.e., physical), while PD in male adolescents related also to more elaborated types (i.e., emotional). This interpretation is only tentative and further research is highly necessary to clarify these observed relationships.

All in all, PD functions as a partial mediator between SV and emotional and physical TDV in boys and between SV and physical, but not emotional, TDV in girls. Importantly, in both cases, boys and girls, these indirect effects are small and the direct effect of SV on TDV remains important and significant after the inclusion of PD in the equation. Given the novel nature of these findings, future research which considered additional third variables is called for.

### 4.4. Limitations and Future Research

Considering that we have been dealing with sensitive information that could be subjected to social desirability (i.e., violence), one limitation of the present study is the use of self-administered anonymous questionnaires as the only source of information. Complementing the study with teacher and peer reports, for instance, might lead to a broader and more comprehensive approach to the topic. A second limitation relates to the relatively small sample size and the information gathered on TDV. We included in our analyses those adolescents who, according to their criteria, had been involved in a romantic relationship within the last year. Further research would probably benefit from gathering additional information (e.g., duration of the relationship, number of dating partners, whether or not dating was occurring now, etc.) so as to be able to refine data analysis. Associated to this and the former limitation, future research might differentiate between those adolescents currently dating, and those who dated in the past, so as to control for potential biases due to retrospective distortion. It is important to note that adolescents in our study were non-clinical, so the range of scores on the measures collected were normative. Especially relevant in the present study is that PD might have not reached clinically significant levels (yet extremely low yet extremely high) and this may have affected our findings of partial mediation. Future research with clinical samples will clarify this issue. Finally, it is worth noting that the use of cross-sectional data poses a statistical limitation, since it is well known that cross-sectional studies are not the most preferable to analyze mediation [85]. Cross-sectional tests of mediation may yield statistical bias that could be solved with longitudinal data sets. So, in order to confirm the relations observed in the present study, longitudinal mediational analysis will have to be conducted which included larger samples, additional sources of information, and which explored alternative relationships among the variables assessed in the present study.

## 5. Conclusions

Despite these and other possible limitations, we highlight the contribution of our findings to the field of TDV. Our results allow advancing in the understanding of the empirical relationship between SV and TDV, which, as evidenced, might be partially explained by PD, especially in male adolescents. If this was replicated in further studies, there are at least two aspects regarding TDV prevention that could be outlined. First, any intervention that targeted TDV should simultaneously focus on the other most important form of violence in adolescents, namely, SV. Second, such intervention should also consider the inclusion of strategies directed to preventing PD so as to neutralize one (likely not the only one) risk factor for TDV.

## Figures and Tables

**Table 1:** Distribution of violence towards a partner by gender, as measured with CADRI.

Form of Violence	Boys	Girls	Total
Emotional (*M* = 18.33; *SD* = 5.45)			
Never	214 (41.2%)	142 (26.5%)	356 (33.7%)
Sometimes	277 (53.4%)	334 (62.3%)	611 (57.9%)
Often	28 (5.4%)	60 (11.2%)	88 (8.3%)
Total	519 (100%)	536 (11.2%)	1055 (100%)
Physical (*M* = 4.46; *SD* = 1.84)			
Never	431 (83.0%)	399 (74.4%)	830 (78.7%)
Sometimes	67 (12.9%)	97 (18.1%)	164 (15.5%)
Often	21 (4.0%)	40 (7.5%)	61 (5.8%)
Total	519 (100%)	536 (100%)	1055 (100%)

Note: The cut-off point between occasional (“sometimes”) and frequent (“often”) violence for emotional and physical violence was 18.33 and 6.30, respectively.

**Table 2:** Spearman significant correlations and descriptive statistics for observable variables for girls (top right) and boys (bottom left).

Variables	1	2	3	4	5	6	7	8	9	10	11	12	13	14	15	16	17	18	19	20	21	22	23	24	25
1. SV1		0.56 **	0.47 **	0.39 **	0.36 **	0.36 **			0.09 *	0.21 **		0.27 **	0.20 **	0.28 **	0.36 **	0.16 **	0.17 **	0.14 **	0.20 **	0.31 **	0.17 **	0.21 **	0.14 **	0.16 **	0.22 **
2. SV2	0.56 **		0.42 **	0.26 **	0.38 **	0.27 **				0.11 *		0.19 **	0.13 **	0.22 **	0.29 **	0.17 **	0.09 *	0.14 **	0.23 **	0.31 **	0.17 **	0.26 **	0.18 **	0.14 **	0.23 **
3. SV3	0.53 **	0.49 **		0.40 **	0.36 **	0.42 **		0.13 **		0.14 **		0.24 **	0.15 **	0.21 **	0.27 **	0.12 **	0.13 **	0.19 **	0.18 **	0.26 **	0.13 **	0.16 **	0.09 *	0.15 **	0.20 **
4. SV4	0.45 **	0.34 **	0.46 **		0.46 **	0.45 **		0.10 *	0.14 **	0.12 **		0.17 **	0.19 **	0.16 **	0.15 **		0.12 **	0.10 *	0.15 **	0.23 **	0.15 **	0.09 *	0.10 *		0.18 **
5. SV5	0.31 **	0.46 **	0.34 **	0.43 **		0.38 **		0.11 *		0.09 *		0.11 **	0.24 **	0.11 *	0.18 **	0.11 *	0.12 **	0.11 **	0.16 **	0.21 **	0.17 **	0.15 **	0.11 *	0.12 **	0.17 **
6. SV6	0.38 **	0.36 **	0.56 **	0.51 **	0.43 **				0.09 *	0.13 **	0.10 *	0.16 **	0.17 **	0.21 **	0.13 **	0.13 **	0.10 *	0.12 **	0.13 **	0.17 **	0.12 **	0.10 *	0.11 *		0.13 **
7. PD1			0.10 *	0.19 **	0.24 **	0.14 **		0.30 **	0.32 **	0.28 **	0.17 **													0.11*	
8. PD2	0.19 **		0.15 **	0.18 **	0.16 **	0.27 **	0.36 **		0.30 **	0.20 **	0.28 **	0.13 **					0.09 *								
9. PD3	0.12 **		0.13 **	0.20 **	0.18 **	0.23 **	0.36 **	0.37 **		0.32 **	0.23 **														
10. PD4	0.12 **	0.12 **	0.19 **	0.22 **	0.22 **	0.25 **	0.35 **	0.30 **	0.37 **		0.33 **	0.18 **	0.13 **	0.18 **	0.13 **	0.09 *		0.11 *							
11. PD5	0.12 **	0.10 *	0.21 **	0.25 **	0.17 **	0.22 **	0.31 **	0.33 **	0.37 **	0.41 **		0.10 *						0.10 *			0.09 *			0.11 *	
12. CAE1	0.15 **	0.09 *	0.16 **	0.15 **	0.15 **	0.13 **		0.13 **	0.09 *	0.11 *			0.29 **	0.37 **	0.30 **	0.17 **		0.18 **	0.26 **	0.41 **	0.25 **	0.26 **	0.16 **	0.19 **	0.23 **
13. CAE2	0.27 **	0.22 **	0.25 **	0.26 **	0.26 **	0.28 **		0.16 **		0.13 **	0.15 **	0.25 **		0.31 **	0.28 **	0.26 **	0.17 **		0.32 **	0.31 **	0.21 **	0.20 **	0.14 **	0.20 **	0.16 **
14. CAE3	0.23 **	0.27 **	0.29 **	0.25 **	0.20 **	0.25 **				0.10 *		0.30 **	0.28 **		0.42 **	0.26 **	0.20 **	0.22 **	0.32 **	0.35 **	0.21 **	0.40 **	0.27 **	0.30 **	0.33 **
15. CAE4	0.15 **	0.15 **	0.23 **	0.18 **	0.17 **	0.19 **			0.09 *	0.09 *		0.20 **	0.34 **	0.35 **		0.42 **	0.20 **	0.16 **	0.34 **	0.32 **	0.29 **	0.31 **	0.22 **	0.32 **	0.29 **
16. CAE5	0.18 **	0.17 **	0.25 **	0.20 **	0.18 **	0.19 **			0.09 *		0.11 *	0.17 **	0.40 **	0.28 **	0.45 **		0.29 **		0.31 **	0.25 **	0.23 **	0.22 **	0.25 **	0.29 **	0.30 **
17. CAE6	0.11 *	0.14 **	0.20 **	0.14 **	0.18 **	0.21 **				0.12 **	0.11 *	0.14 **	0.28 **	0.26 **	0.27 **	0.53 **			0.27 **	0.12 **	0.16 **	0.19 **	0.36 **	0.22 **	0.18 **
18. CAE7	0.09 *	0.09 *	0.18 **	0.22 **	0.17 **	0.24 **	0.13 **	0.10 *	0.13 **		0.13 **	0.23 **	0.29 **	0.20 **	0.28 **	0.27 **	0.27 **		0.16 **	0.24 **	0.18 **	0.12 **		0.21 **	0.23 **
19. CAE8	0.17 **	0.17 **	0.22 **	0.16 **	0.17 **	0.23 **		0.10 *	0.17 **	0.09 *	0.12 **	0.28 **	0.41 **	0.31 **	0.35 **	0.44 **	0.28 **	0.31 **		0.37 **	0.29 **	0.19 **	0.19 **	0.23 **	0.18 **
20. CAE9	0.12 **		0.14 **	0.17 **	0.14 **	0.15 **	0.10 *	0.19 **	0.12 **		0.09 *	0.30 **	0.28 **	0.28 **	0.31 **	0.21 **	0.15 **	0.23 **	0.36 **		0.34 **	0.23 **	0.17 **	0.20 **	0.23 **
21. CAE10	0.19 **	0.15 **	0.28 **	0.25 **	0.16 **	0.29 **	0.13 **	0.14 **	0.11 *	0.15 **	0.12 **	0.20 **	0.32 **	0.27 **	0.33 **	0.41 **	0.39 **	0.39 **	0.35 **	0.30 **		0.23 **	0.10 *	0.20 **	0.16 **
22. CAP1	0.14 **	0.22 **	0.27 **	0.17 **	0.13 **	0.22 **		0.11 *	0.16 **	0.18 **	0.09 *	0.14 **	0.25 **	0.29 **	0.25 **	0.34 **	0.36 **	0.20 **	0.27 **	0.13 **	0.28 **		0.34 **	0.30 **	0.35 **
23. CAP2		0.12 **	0.14 **	0.11 *	0.09 *	0.19 **			0.10 *		0.12 **	0.10 *	0.22 **	0.13 **	0.22 **	0.38 **	0.37 **	0.23 **	0.19 **		0.26 **	0.32 **		0.34 **	0.39 **
24. CAP3	0.14**	0.16 **	0.25 **	0.18 **	0.16 **	0.25 **			0.12 **		0.09 *	0.18 **	0.29 **	0.23 **	0.25 **	0.39 **	0.40 **	0.33 **	0.31 **	0.17 **	0.33 **	0.35 **	0.42 **		0.46 **
25. CAP4	0.13**	0.12 **	0.17 **	0.18 **	0.10 *	0.16 **					0.12 **		0.32 **	0.14 **	0.27 **	0.44 **	0.28 **	0.25 **	0.25 **	0.15 **	0.29 **	0.37 **	0.37 **	0.53 **	
Mean boys	5.68	6.80	5.86	5.29	7.08	4.88	2.44	2.29	2.18	2.03	1.89	1.41	1.24	1.45	1.24	1.11	1.09	1.15	1.21	1.26	1.14	1.21	1.05	1.06	1.04
SD boys	1.80	2.74	1.86	1.63	2.23	1.62	1.24	1.16	1.14	1.19	1.13	0.66	0.57	0.77	0.55	0.39	0.34	0.48	0.51	0.57	0.47	0.57	0.28	0.31	0.27
Mean girls	5.54	5.73	5.60	5.44	7.39	4.74	2.86	2.51	2.62	2.30	2.07	1.73	1.36	1.46	1.47	1.14	1.08	1.14	1.33	1.57	1.26	1.30	1.09	1.08	1.10
SD girls	1.61	2.17	1.44	1.64	2.15	1.44	1.29	1.18	1.29	1.21	1.15	0.85	0.69	0.77	0.77	0.47	0.30	0.45	0.63	0.83	0.59	0.64	0.35	0.38	0.40

Note: SV = School Violence; PD = Personal Distress; CAE = Conflict in Adolescent Dating Relationships: Emotional; CAP = Conflict in Adolescent Dating Relationships: Physical; * *p* < 0.05; ** *p* < 0.01.

**Table 3:** Factor loadings, Cronbach’s Alpha, McDonald’s omega coefficients and covariances between latent variables on the measurement model for boys/girls.

Item	Factor Loading	Cronbach’s Alpha	McDonald’s Omega	Latent Variable	Covariance		
					Personal Distress	Conflict: Emotional	Conflict: Physical
SV1	0.80 **/0.60 **	0.84/0.83	0.86/0.85	School Violence	0.41 **/0.18 **	0.48 **/0.45 **	0.47 **/0.46 **
SV2	0.74 **/0.83 **						
SV3	0.65 **/0.67 **						
SV4	0.80 **/0.69 **						
SV5	0.76 **/0.66 **						
SV6	0.64 **/0.64 **						
PD1	0.51 **/0.41 **	0.75/0.66	0.75/0.66	Personal Distress	-	0.33 **/0.20 **	0.36 */0.13
PD2	0.64 **/0.42 **						
PD3	0.57 **/0.45 **						
PD4	0.66 **/0.83 **						
PD5	0.65 **/0.46 **						
CAE1	0.50 **/0.38 **	0.79/0.79	0.80/0.80	Conflict: Emotional	-	-	0.89 **/0.87 **
CAE2	0.48 **/0.63 **						
CAE3	0.71 **/0.67 **						
CAE4	0.66 **/0.74 **						
CAE5	0.66 **/0.77 **						
CAE6	0.83 **/0.44 **						
CAE7	0.83 **/0.69 **						
CAE8	0.75 **/0.57 **						
CAE9	0.72 **/0.74 **						
CAE10	0.89 **/0.84 **						
CAP1	0.78 **/0.64 **	0.66/0.70	0.730/.76	Conflict: Physical	-	-	-
CAP2	0.75 **/0.71 **						
CAP3	0.61 **/0.72 **						
CAP4	0.86 **/0.61 **						

Note: SV = School Violence; PD = Personal Distress; CAE = Conflict in Adolescent Dating Relationships: Emotional; CAP = Conflict in Adolescent Dating Relationships: Physical. Factor loadings and covariances between latent variables are standardized values; * *p* < 0.05; ** *p* < 0.01.

**Table 4:** Fit indices and path coefficients for the mediation model for boys.

Cond.	Model	Fit Index								Path Coefficient			
		χ^2^	*df*	RMSEA	90 %CI		CFI	TLI	SRMR	Relation	β	95% CI	
					LL	UL						LL	UL
1	A-C	204.56 *	167	0.02	0.01	0.03	0.98	0.98	0.06	A-C1	0.47 **	0.45	0.49
										A-C2	0.49 **	0.47	0.50
2	Cons. A-B-C	340.88 **	271	0.02	0.01	0.03	0.96	0.96	0.07	A-B	0.71 **	0.66	0.74
										B-C1	0.65 **	0.55	0.67
										B-C2	0.64 **	0.61	0.68
3	Cons. A-B-C	340.88 **	271	0.02	0.01	0.03	0.96	0.96	0.07	-	-	-	-
	Uncons. A-B-C	308.90 *	269	0.02	0.00	0.03	0.98	0.98	0.06	A-B	0.42 **	0.35	0.48
										A-C1	0.41 **	0.37	0.45
										A-C2	0.41 **	0.34	0.45
										B-C1	0.15 **	0.01	0.24
										B-C2	0.19 **	0.11	0.32
	Diff test	25.13 **	2	-	-	-	-	-		-	-	-	-
4	Indirect effect test	-	-	-	-	-	-	-		A-C1	0.06 *	0.00	0.11
										A-C2	0.08 **	0.03	0.13

Note: Cond. = Casual step condition; *df* = degrees of freedom; RMSEA = Root mean square error of approximation; CFI = Comparative fit index; TLI: Tucker-Lewis index; SRMR = Standardized root mean square residual; CI = Confidence interval; LL = Lower limit; UL = Upper limit; Cons. = Constrained; Uncons. = Unconstrained; Diff. test = Loglikelihood Chi Square Difference Test; A = Independent variable (School Violence, SV); B = Mediator (Personal Distress, PD); C = Dependent variables, C1 (Conflict in Adolescent Dating Relationships Inventory: Emotional, CAE), C2 (Conflict in Adolescent Dating Relationships Inventory: Physical, CAP); * *p* < 0.05; ** *p* < 0.01.

**Table 5:** Fit indices and path coefficients for the mediation model for girls.

Cond.	Model	Fit Index								Path Coefficient			
		χ^2^	*df*	RMSEA	90% CI		CFI	TLI	SRMR	Relation	β	95% CI	
					LL	UL						LL	UL
1	A-C	231.51 **	167	0.03	0.02	0.04	0.97	0.97	0.07	A-C1	0.42 **	0.24	0.60
										A-C2	0.42 **	0.23	0.57
2	Cons. A-B-C	384.25 **	271	0.03	0.02	0.03	0.94	0.93	0.08	A-B	0.58 **	0.38	0.75
										B-C1	0.72 **	0.06	0.83
										B-C2	0.69 **	0.61	0.78
3	Cons. A-B-C	384.25 **	271	0.03	0.02	0.03	0.94	0.93	0.08	-			
	Uncons. A-B-C	344.12 **	269	0.02	0.02	0.03	0.96	0.96	0.07	A-B	0.18 **	0.06	0.26
										A-C1	0.40 **	0.23	0.59
										A-C2	0.40 **	0.22	0.57
										B-C1	0.15 **	0.05	0.24
										B-C2	0.10 **	0.03	0.16
	Diff test	64.85 **	2	-	-	-	-	-		-			
4	Indirect effect test	-	-	-	-	-	-	-		A-C1	0.03	0.01	0.06
										A-C2	0.02 *	0.01	0.03

Note: Cond. = Casual step condition; *df* = degrees of freedom; RMSEA = Root mean square error of approximation; CFI = Comparative fit index; TLI: Tucker-Lewis index; SRMR = Standardized root mean square residual; CI = Confidence interval; LL = Lower limit; UL = Upper limit; Cons. = Constrained; Uncons. = Unconstrained; Diff. test = Loglikelihood Chi Square Difference Test; A = Independent variable (School Violence, SV); B = Mediator (Personal Distress, PD); C = Dependent variables, C1 (Conflict in Adolescent Dating Relationships Inventory: Emotional, CAE), C2 (Conflict in Adolescent Dating Relationships Inventory: Physical, CAP); * *p* < 0.05; ** *p* < 0.01.

## Appendix A

**Table A1:** Spearman correlations and descriptive statistics for observable variables for girls (top right) and boys (bottom left). Complete table.

Variables	1	2	3	4	5	6	7	8	9	10	11	12	13	14	15	16	17	18	19	20	21	22	23	24	25
1. SV1		0.56 **	0.47 **	0.39 **	0.36 **	0.36 **	−0.1	0.07	0.09 *	0.21 **	0.02	0.27 **	0.20 **	0.28 **	0.36 **	0.16 **	0.17 **	0.14 **	0.20 **	0.31 **	0.17 **	0.21 **	0.14 **	0.16 **	0.22 **
2. SV2	0.56 **		0.42 **	0.26 **	0.38 **	0.27 **	−0.03	0.02	0.02	0.11 *	−0.03	0.19 **	0.13 **	0.22 **	0.29 **	0.17 **	0.09 *	0.14 **	0.23 **	0.31 **	0.17 **	0.26 **	0.18 **	0.14 **	0.23 **
3. SV3	0.53 **	0.49 **		0.40 **	0.36 **	0.42 **	0.00	0.13 **	0.07	0.14 **	0.03	0.24 **	0.15 **	0.21 **	0.27 **	0.12 **	0.13 **	0.19 **	0.18 **	0.26 **	0.13 **	0.16 **	0.09 *	0.15 **	0.20 **
4. SV4	0.45 **	0.34 **	0.46 **		0.46 **	0.45 **	0.06	0.10 *	0.14 **	0.12 **	0.04	0.17 **	0.19 **	0.16 **	0.15 **	0.06	0.12 **	0.10 *	0.15 **	0.23 **	0.15 **	0.09 *	0.10 *	0.08	0.18 **
5. SV5	0.31 **	0.46 **	0.34 **	0.43 **		0.38 **	0.05	0.11 *	0.08	0.09 *	0.07	0.11 **	0.24 **	0.11 *	0.18 **	0.11 *	0.12 **	0.11 **	0.16 **	0.21 **	0.17 **	0.15 **	0.11 *	0.12 **	0.17 **
6. SV6	0.38 **	0.36 **	0.56 **	0.51 **	0.43 **		0.01	0.06	0.09 *	0.13 **	0.10 *	0.16 **	0.17 **	0.21 **	0.13 **	0.13 **	0.10 *	0.12 **	0.13 **	0.17 **	0.12 **	0.10 *	0.11 *	0.07	0.13 **
7. PD1	0.06	0.04	0.10 *	0.19 **	0.24 **	0.14 **		0.30 **	0.32 **	0.28 **	0.17 **	0.01	−0.08	0.02	−0.02	0.05	0.05	0.00	−0.01	−0.04	0.00	0.02	0.03	0.11 *	0.00
8. PD2	0.19 **	0.06	0.15 **	0.18 **	0.16 **	0.27 **	0.36 **		0.30 **	0.20 **	0.28 **	0.13 **	0.08	0.04	0.01	0.05	0.09 *	0.05	0.08	0.05	0.04	0.01	0.00	−0.02	0.04
9. PD3	0.12 **	0.03	0.13 **	0.20 **	0.18 **	0.23 **	0.36 **	0.37 **		0.32 **	0.23 **	0.00	0.05	0.03	−0.01	0.00	−0.02	0.02	0.05	0.03	0.01	−0.01	−0.02	−0.01	0.04
10. PD4	0.12 **	0.12 **	0.19 **	0.22 **	0.22 **	0.25 **	0.35 **	0.30 **	0.37 **		0.33 **	0.18 **	0.13 **	0.18 **	0.13 **	0.09 *	0.08	0.11 *	0.07	0.04	0.03	0.07	0.07	0.09	0.07
11. PD5	0.12 **	0.10 *	0.21 **	0.25 **	0.17 **	0.22 **	0.31 **	0.33 **	0.37 **	0.41 **		0.10 *	0.08	0.04	0.03	0.00	−0.01	0.10 *	0.02	0.06	0.09 *	0.01	−0.02	0.11 *	0.02
12. CAE1	0.15 **	0.09 *	0.16 **	0.15 **	0.15 **	0.13 **	0.02	0.13 **	0.09 *	0.11 *	0.07		0.29 **	0.37 **	0.30 **	0.17 **	0.08	0.18 **	0.26 **	0.41 **	0.25 **	0.26 **	0.16 **	0.19 **	0.23 **
13. CAE2	0.27 **	0.22 **	0.25 **	0.26 **	0.26 **	0.28 **	0.08	0.16 **	0.09	0.13 **	0.15 **	0.25 **		0.31 **	0.28 **	0.26 **	0.17 **	0.08	0.32 **	0.31 **	0.21 **	0.20 **	0.14 **	0.20 **	0.16 **
14. CAE3	0.23 **	0.27 **	0.29 **	0.25 **	0.20 **	0.25 **	0.08	0.08	0.06	0.10 *	0.08	0.30 **	0.28 **		0.42 **	0.26 **	0.20 **	0.22 **	0.32 **	0.35 **	0.21 **	0.40 **	0.27 **	0.30 **	0.33 **
15. CAE4	0.15 **	0.15 **	0.23 **	0.18 **	0.17 **	0.19 **	0.06	0.06	0.09 *	0.09 *	0.06	0.20 **	0.34 **	0.35 **		0.42 **	0.20 **	0.16 **	0.34 **	0.32 **	0.29 **	0.31 **	0.22 **	0.32 **	0.29 **
16. CAE5	0.18 **	0.17 **	0.25 **	0.20 **	0.18 **	0.19 **	0.02	0.07	0.09 *	0.06	0.11 *	0.17 **	0.40 **	0.28 **	0.45 **		0.29 **	0.05	0.31 **	0.25 **	0.23 **	0.22 **	0.25 **	0.29 **	0.30 **
17. CAE6	0.11 *	0.14 **	0.20 **	0.14 **	0.18 **	0.21 **	0.01	0.09	0.08	0.12 **	0.11 *	0.14 **	0.28 **	0.26 **	0.27 **	0.53 **		0.06	0.27 **	0.12 **	0.16 **	0.19 **	0.36 **	0.22 **	0.18 **
18. CAE7	0.09 *	0.09 *	0.18 **	0.22 **	0.17 **	0.24 **	0.13 **	0.10 *	0.13 **	0.08	0.13 **	0.23 **	0.29 **	0.20 **	0.28 **	0.27 **	0.27 **		0.16 **	0.24 **	0.18 **	0.12 **	0.06	0.21 **	0.23 **
19. CAE8	0.17 **	0.17 **	0.22 **	0.16 **	0.17 **	0.23 **	0.06	0.10 *	0.17 **	0.09 *	0.12 **	0.28 **	0.41 **	0.31 **	0.35 **	0.44 **	0.28 **	0.31 **		0.37 **	0.29 **	0.19 **	0.19 **	0.23 **	0.18 **
20. CAE9	0.12 **	0.09	0.14 **	0.17 **	0.14 **	0.15 **	0.10 *	0.19 **	0.12 **	0.06	0.09 *	0.30 **	0.28 **	0.28 **	0.31 **	0.21 **	0.15 **	0.23 **	0.36 **		0.34 **	0.23 **	0.17 **	0.20 **	0.23 **
21. CAE10	0.19 **	0.15 **	0.28 **	0.25 **	0.16 **	0.29 **	0.13 **	0.14 **	0.11 *	0.15 **	0.12 **	0.20 **	0.32 **	0.27 **	0.33 **	0.41 **	0.39 **	0.39 **	0.35 **	0.30 **		0.23 **	0.10 *	0.20 **	0.16 **
22. CAP1	0.14 **	0.22 **	0.27 **	0.17 **	0.13 **	0.22 **	0.03	0.11 *	0.16 **	0.18 **	0.09 *	0.14 **	0.25 **	0.29 **	0.25 **	0.34 **	0.36 **	0.20 **	0.27 **	0.13 **	0.28 **		0.34 **	0.30 **	0.35 **
23. CAP2	0.08	0.12 **	0.14 **	0.11 *	0.09 *	0.19 **	0.02	0.05	0.10 *	0.05	0.12 **	0.10 *	0.22 **	0.13 **	0.22 **	0.38 **	0.37 **	0.23 **	0.19 **	0.03	0.26 **	0.32 **		0.34 **	0.39 **
24. CAP3	0.14 **	0.16 **	0.25 **	0.18 **	0.16 **	0.25 **	0.02	0.06	0.12 **	0.08	0.09 *	0.18 **	0.29 **	0.23 **	0.25 **	0.39 **	0.40 **	0.33 **	0.31 **	0.17 **	0.33 **	0.35 **	0.42 **		0.46 **
25. CAP4	0.13 **	0.12 **	0.17 **	0.18 **	0.10 *	0.16 **	−0.01	0.06	0.08	0.01	0.12 **	0.05	0.32 **	0.14 **	0.27 **	0.44 **	0.28 **	0.25 **	0.25 **	0.15 **	0.29 **	0.37 **	0.37 **	0.53 **	
Mean boys	5.68	6.80	5.86	5.29	7.08	4.88	2.44	2.29	2.18	2.03	1.89	1.41	1.24	1.45	1.24	1.11	1.09	1.15	1.21	1.26	1.14	1.21	1.05	1.06	1.04
SD boys	1.80	2.74	1.86	1.63	2.23	1.62	1.24	1.16	1.14	1.19	1.13	0.66	.57	0.77	0.55	0.39	0.34	0.48	0.51	0.57	0.47	0.57	0.28	0.31	0.27
Mean girls	5.54	5.73	5.60	5.44	7.39	4.74	2.86	2.51	2.62	2.30	2.07	1.73	1.36	1.46	1.47	1.14	1.08	1.14	1.33	1.57	1.26	1.30	1.09	1.08	1.10
SD girls	1.61	2.17	1.44	1.64	2.15	1.44	1.29	1.18	1.29	1.21	1.15	0.85	0.69	0.77	0.77	0.47	0.30	0.45	0.63	0.83	0.59	0.64	0.35	0.38	0.40

Note: SV = School Violence; PD = Personal Distress; CAE = Conflict in Adolescent Dating Relationships: Emotional; CAP = Conflict in Adolescent Dating Relationships: Physical; * *p* < 0.05; ** *p* < 0.01.
